# Hopelessness, Dissociative Symptoms, and Suicide Risk in Major Depressive Disorder: Clinical and Biological Correlates

**DOI:** 10.3390/brainsci10080519

**Published:** 2020-08-05

**Authors:** Mauro Pettorruso, Giacomo d’Andrea, Giovanni Martinotti, Fabrizio Cocciolillo, Andrea Miuli, Ilenia Di Muzio, Rebecca Collevecchio, Valeria Verrastro, Fabio De-Giorgio, Luigi Janiri, Massimo di Giannantonio, Daniela Di Giuda, Giovanni Camardese

**Affiliations:** 1 Department of Neuroscience, Imaging and Clinical Sciences, “G. d’Annunzio” University, Via dei Vestini 33, 66013 Chieti, Italy; mauro.pettorruso@unich.it (M.P.); giovanni.martinotti@gmail.com (G.M.); andreamiuli@live.it (A.M.); dimuzioilenia@gmail.com (I.D.M.); rebeccacollevecchio@gmail.com (R.C.); digiannantonio@unich.it (M.d.G.); 2 Department of Pharmacy, Pharmacology, Clinical Science, University of Hertfordshire, Herts AL109AB, UK; 3 Unità Operativa Complessa di Medicina Nucleare, Fondazione Policlinico Universitario A. Gemelli IRCCS, 00168 Roma, Italia; f.cocciolillo@gmail.com (F.C.); daniela.digiuda@unicatt.it (D.D.G.); 4 Istituto di Medicina Nucleare, Università Cattolica del Sacro Cuore, 00168 Roma, Italia; 5 Department of Medical and Surgical Sciences, Magna Graecia University of Catanzaro, 88100 Catanzaro, Italy; Valeriaverrastro@istitutopsicoterapie.it; 6 Section of Legal Medicine, Institute of Public Health, Università Cattolica del Sacro Cuore, 00168 Rome, Italy; fabiodegiorgio3@gmail.com; 7 Department of Psychiatry, Fondazione Policlinico Universitario “A. Gemelli” IRCCS, 00168 Rome, Italy; luigi.janiri@unicatt.it (L.J.); giovanni.camardese@unicatt.it (G.C.); 8 Institute of Psychiatry, Università Cattolica del Sacro Cuore, 00168 Rome, Italy

**Keywords:** dopamine transporter, suicidality, mood disorders, basal ganglia, DaTSCAN, dissociation

## Abstract

*Background:* Major depressive disorder (MDD) has different clinical presentations and is associated with neurobiological alterations. Hopelessness, anhedonia, and dissociation represent some of the most pervasive psychopathological symptoms that often lead to suicidal thoughts, attempts, and actions. To further research on the concept of depression endophenotypes, this study aimed to assess the possible relationships between hopelessness and other clinical and biological correlates (i.e., striatal dopaminergic dysfunction) in depressed patients. *Methods:* We recruited 51 subjects with MDD. All subjects underwent ^123^I-FP-CIT SPECT to assess striatal dopamine transporter (DAT) availability and a psychometric evaluation using the psychometric scale to assess depressive, anxious, dissociative, and hopelessness symptoms aside from suicidal ideation. *Result:* An inverse correlation between the hopelessness score and dopamine transporter availability in all basal ganglia was bilaterally found. (Right Putamen, *r* = −0.445, *p* < 0.01; Left Putamen, *r* = −0.454, *p* < 0.01; Right Caudate, *r* = −0.398, *p* < 0.01; Left Caudate, *r* = −0.467, *p* < 0.01) Moreover, a positive correlation was also found between hopelessness and dissociative symptoms. *Conclusions:* These results provide important evidence on the neurobiological and clinical correlates of different psychopathological symptoms of depression with potential implications in terms of devising more effective treatment programs.

## 1. Introduction

Major depressive disorder (MDD) is a high-burden disease characterized by several psychopathological dimensions, including mood deflection, suicidality, psychomotor retardation or agitation, loss of motivation, hopelessness and anhedonia [1]. In recent decades, hopelessness has emerged as one of the core features of MDD [2]. It could be defined as a dimension characterized by negative expectancies for the future, lack of general motivation, and the attribution of wrong meanings to personal experiences [3]. From a clinical point of view, the experience of a lack of positive expectations for the future combined with the distress and mental pain that often occurs in depressed patients leads to an increased risk of suicide, as it appears the only way to escape from their inner “unsolvable problems.” In fact, hopelessness seems to be strictly related to suicidal ideation and this relationship is stronger than the one between suicidality and depression severity [4], as reported by a recent systematic review on this argument [2]. In addition, hopelessness could be considered a clinical predictor for any suicide attempts [5] (with a 90–94.2% accuracy [6,7]) even when depressive symptoms are contained [8].

In addition to the dimensions classically related to MDD and suicide, (i.e., impulsivity, helplessness, mental pain, rumination) dissociative symptoms seem to play a leading role in the psychopathological processes of depression [9,10]. In this clinical context, dissociative symptoms seem to be attempts to isolate particular mental processes, such as thoughts, feelings, or states of mind that could be too distressing or painful to be mentalized from consciousness [11]. This seems to be particularly true for patients who suffer hopelessness, who find a way to deal with the lack of positive future prospects through dissociation [12].

In recent years, studies have attempted to explain the psychopathological aspects of MDD through investigating its neurobiological underpinnings to identify dimension-targeted pharmacological treatments. Based on the historical monoamine hypothesis of depression [13], the leading roles of serotonin (5-HT), norepinephrine (NE), and dopamine (DA) in the pathophysiology of MDD are well-known. Nowadays, there is an increasing interest in the role of the dopaminergic synapses in MDD, as confirmed by the numerous preclinical, neuroimaging, and pharmacological studies on this subject [14]. The dopamine transporter (DAT) represents an important element of the synaptic cleft and regulates DA activities through the presynaptic reuptake of the neurotransmitter in the striatum and midbrain. It is often used as a target for the evaluation of dopaminergic circuit efficiency [15]. Using peculiar radiotracers for DAT, single-photon emission computerized tomography (SPECT) has been extensively used in MDD patients and has shown an important reduction in DAT availability in subcortical regions as result of a striatal dopaminergic dysfunction [16,17,18]. In particular, several studies have focused on the roles of this dysfunction in anhedonic patients with MDD by following the hypothesis of a cortico-striatal-limbic dopaminergic dysfunction, involving selected areas of ventral (nucleus accumbens) and dorsal (putamen, caudate) striatum, in which the reward system seems to play a crucial role [19,20]. One of the clinical correlates of reward system disfunction appears to be the lack of motivation, a core element of both anhedonia and hopelessness.

Concerning suicidality, the roles of dopaminergic dysregulations are still relatively unknown [21] since the literature has reported contrasting results in both in vivo and post-mortem analysis. On one hand, some studies showed diminished striatal dopaminergic signaling in depressed patients attempting suicide [22,23]. On the other hand, several studies failed to find a correlation between suicidality and striatal dopaminergic dysfunctions [24,25,26].

Considering the common psychopathological aspects shared by anhedonia and hopelessness, such as the loss of motivation and negative beliefs, which seem to involve the dopaminergic reward system, the main aim of this study was to assess whether hopelessness is related to a peculiar striatal dopaminergic dysfunction in a sample of depressed patients; in addition, we assess suicidality through the use of a single item of Hamilton Depression Rating Scale, in order to evaluate a possible correlation between suicide risk and DAT availability. Secondarily, we investigated possible relationships between hopelessness and other psychopathological dimensions of MDD to assess their clinical impact on suicidality.

## 2. Materials and Methods

### 2.1. Subjects

Patients enrolled at the “A. Gemelli” Hospital of the Catholic University of the Sacred Heart in Rome, Italy were considered for this study. All subjects underwent ^123^I-FP-CIT SPECT to assess striatal DAT availability, which was performed at the Nuclear Medicine Unit of the Catholic University of the Sacred Heart. Only patients with a current major depressive episode (MDE) in the context of MDD following the Diagnostic and Statistical Manual of Mental Disorders (DSM-5) criteria were extracted retrospectively from a previously collected dataset. We included in the study subjects not reporting SPECT alteration according to BASAL GANGLIA software [27] and without any neurological diagnosis in the subsequent three years of clinical follow-up. An accurate neurological examination was performed to rule out idiopathic Parkinson’s disease. Exclusion criteria included the diagnosis of Parkinson’s disease or Atypical Parkinsonism, past or current abuse or substance/alcohol use disorders, bipolar or psychotic disorders, and the current use of psychoactive medication with action towards the dopaminergic system.

The sample size was established on the basis of a power analysis with a minimum Rho correlation set to 0.4, power value was 0.80 with a type one error rate (alpha) set to 0.05 in the current sample.

Fifty-one depressed patients were enrolled.

Before the diagnostic exams, all patients were evaluated through a clinical interview conducted by trained medical staff. During this interview, some psychometric scales largely and widely approved by the literature were used; these scales included the 21-item Hamilton Depression Rating Scale (HDRS-21) (Cronbach alpha value = 0.7) [28] to assess depression severity, the Beck Hopelessness Scale (BHS) to evaluate hopelessness (Cronbach alpha value = 0.87) [29], the Hamilton Anxiety Rating Scale (HARS) to assess anxiety symptoms (Cronbach alpha value = 0.89) [30] and the Dissociative Experiences Scale (DES) to screen dissociative symptoms (Cronbach alpha value = 0.96) [31]. In order to evaluate suicidality, we used the third item of the HDRS-21, which investigates the intensity of suicidal thoughts (with a range from 0 to 4).

The study was approved by the ethical committee of the “Università Cattolica del Sacro Cuore” (Protocol Number: 16113/13, date: 17 July 2013), all patient data were treated confidentially and anonymously, and the interviews were conducted in line with the Helsinki Declaration (2013) [32].

### 2.2. SPECT Procedure

SPECT acquisition and processing were performed as previously published [16,33].

All subjects had been free from psychotropic drugs for 4 weeks before the SPECT study to adhere to the current neuroimaging guidelines. [34] Thirty minutes after the thyroid blockade, which was administered using 400 mg of oral potassium perchlorate, an intravenous injection of 185 MBq of ^123^I-FP-CIT was given (DaTSCAN, GE Healthcare).

SPECT was carried out using a dual-head gamma camera system (E.CAM, Siemens Medical System) equipped with high resolution, low-energy, parallel-hole collimators.

Acquisition started between 180 and 240 min after injection and lasted 45 min.

We applied a “step-and-shoot” protocol with a radius of rotation ≤15 cm and the following parameters: 120 projection angles over 360° (angular step of 3°), 128 × 128 matrix, 1.23 zoom factor, 3.90 × 3.90 mm pixel size, and 45 s per view. Data were reconstructed using filtered back projection using a Butterworth filter (cut-off frequency: 0.45 cycle/cm, order: 8). Chang’s first-order attenuation correction was also applied (attenuation coefficient: 0.11 cm^−1^). Reconstructed transaxial, sagittal, and coronal slices (slice thickness: 3.9 mm) were reoriented according to the canthomeatal plane. Data were collected and analyzed by the same observer, who was blind to all clinical information and psychometric measures. After preliminary qualitative (visual) assessments, region of interest (ROI) analyses were performed to quantify the striatal 123I-FP-CIT binding ratios. Five adjacent transaxial slices representing the most intense striatal uptake were summed up in a single 19.5-mm-thick reference section. A set of two-dimensional ROIs was manually drawn with the help of an anatomical brain atlas [35] and saved as a template. The following ROIs were positioned on the reference section: two symmetrical irregular ROIs over the right and left striata (as radiotracer-specific binding), two symmetrical circular ROIs over the right and left caudates and putamen, respectively (as radiotracer-specific binding), and one irregular ROI over the occipital cortex (as radiotracer non-specific binding).

All ROIs were positioned by the same highly experienced observer. The mean counts per pixel in each region were used for semi-quantitative analysis. Specific and non-specific 123I-FP-CIT binding ratios were calculated bilaterally for the striatum, putamen, and caudate nucleus using the following formula: ((mean counts in striatal ROI)-(mean counts in occipital ROI))/(mean counts in occipital ROI).

### 2.3. Statistical Analysis

Statistical analysis was performed using SPSS for MAC 26.0 (SPSS inc. Chicago, Illinois) software. Continuous variables were expressed as a mean ± standard deviation (SD), while categorical variables are reported as average number and percentage. Pearson’s correlation analysis was used to assess the relationship between SPECT data and psychometric measures. A Multiple linear regression has been conducted to investigate the potential impact of confounding variables (i.e., sex, age) on changes in striatal DAT availability.

Two groups (high suicide risk vs. low suicide risk) were created retrospectively based on the results obtained at the 3rd item of the HDRS-21 on suicide. The two groups were compared through the ANOVA analysis.

All tests were two-sided with a level of significance set at *p* < 0.05.

## 3. Results

### 3.1. Sociodemographic and Clinical Assessment

The total number of subjects enrolled in this study was 51 (22 males; 43.1%), with a mean age of 65.82 ± 12.42. All sociodemographic and psychometric data are reported in Table 1. Striatal DAT availability is reported in Table 2.

### 3.2. Correlations between Psychometric Scales and Striatal DAT Availability

All variables were normally distributed according to the Shapiro–Wilk test.

Pearson correlation analyses were conducted to investigate possible correlations between the specific dimensions evaluated through psychometric assessments and the DAT availability in the basal ganglia. According to our analysis, depression severity showed an inverse correlation with DAT availability in right Caudate (*R* = −0.388, *p* = 0.005) and Left Putamen (*R* = −0.291, *p* = 0.038).

**Figure 1:**
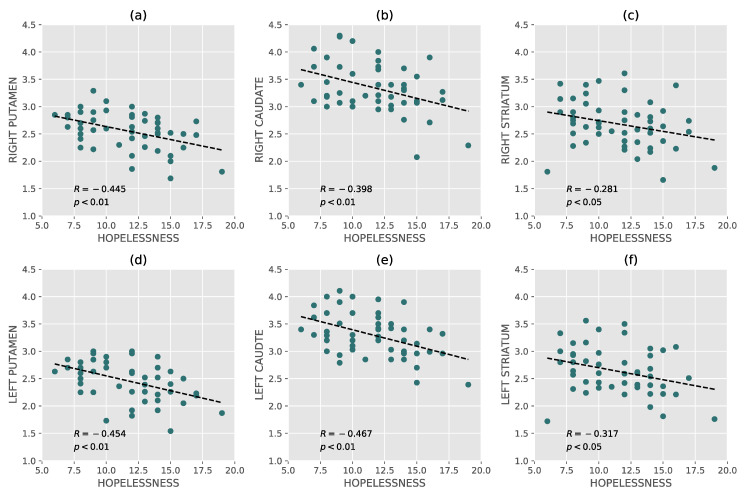
Correlations between hopelessness and dopamine transporter. (**a**): Correlation between hopelessness and right putamen. (**b**): Correlation between hopelessness and right caudate. (**c**): Correlation between hopelessness and right striatum. (**d**): Correlation between hopelessness and left putamen. (**e**): correlation between hopelessness and left caudate. (**f**): Correlation between hopelessness and left striatum. The correlation coefficients and p-values are reported for each plot.

In addition, an inverse correlation was found between hopelessness and DAT availability in all basal ganglia areas bilaterally, showing that as the hopelessness intensity in depressed patients increased, DAT levels in the basal ganglia reduced progressively, as shown in Figure 1 (all correlation coefficients and confidence intervals are available in Supplementary Materials).

A multiple linear regression has been conducted to investigate the potential impact of confounding variables (i.e., sex, age) on the association between hopelessness and striatal DAT availability in our sample.

Hopelessness was the only factor associated to striatal DAT modifications in bilateral putamen and left caudate (right putamen: BHS beta = −0.457, *p* = 0.001; Sex. Beta = 0.001, *p* = 0.994; Age beta = 0.114, *p* = 0.403; left putamen BHS beta = −0.462, *p* = 0.001; Sex. Beta = 0.057, *p* = 0.673; Age beta = 0.077, *p* = 0.569; left caudate BHS beta = −0.489, *p* < 0.001; Sex. Beta = 0.130, *p* = 0.317; Age beta = 0.228, *p* = 0.085; left striatum BHS beta = −0.327, *p* = 0.021; Sex. Beta = −0.094, *p* = 0.509; Age beta = 0.99, *p* = 0.489; right striatum BHS beta = −0.290, *p* = 0.043; Sex. Beta = −0.068, *p* = 0.640; Age beta = 0.088, *p* = 0.544), while a simultaneous role of age was detected in right caudate region (right caudate BHS beta = −0.425, *p* = 0.002; Sex. Beta = 0.238, *p* = 0.074; Age beta = 0.277, *p* = 0.040).

No other significant correlations between psychometric scores and DAT availability were found (all correlation coefficients and confidence intervals are available in Supplementary Materials).

### 3.3. Suicide Risk and Striatal DAT Availability

High suicide risk group showed lower DAT availability in Left Putamen and Right Caudate, compaired to low suicide risk group, as reported in Table 3.

### 3.4. Correlations between Psychometric Scales

**Figure 2:**
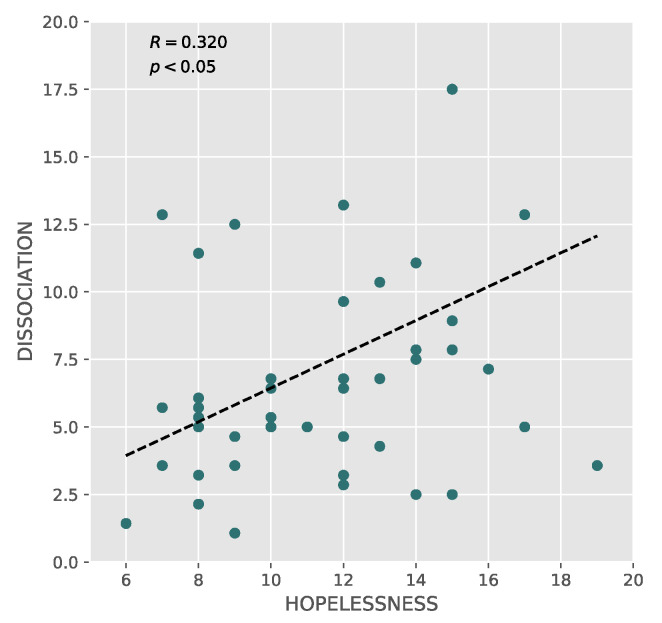
Correlations between hopelessness and dissociation.

We found a significant positive correlation between hopelessness and dissociation (Pearson’s correlation analysis: *r* = 0.320, *p* = 0.05; Figure 2), reflecting an increase in dissociative symptoms in depressed patients with higher hopelessness, as shown in Figure 2.

In addition, depressive symptomatology intensity evaluated through the HDRS-21 was significantly correlated to dissociation (Pearson’s correlation analysis: *r* = 0.386; *p* = 0.01) and hopelessness (Pearson’s correlation analysis: *r* = 0.371, *p* = 0.01). All other correlation analyses between psychometric scales are reported in the Supplementary Materials.

## 4. Discussion

Our results show the existence of an inverse correlation between hopelessness and DAT availability, which appears to have widely decreased in all basal ganglia. To the best of our knowledge, this is the first study to show a relationship between hopelessness in MDD and striatal dopaminergic dysfunction. 

The existence of a striatal DA dysfunction has been highlighted in several preclinical and clinical studies on MDD [14]. These findings seem to be particularly consistent for depressed patients with specific psychopathological characteristics, such as anhedonia, deeply related to the dopaminergic reward system [20,36]. Previous studies, in fact, have found a dopaminergic striatal dysfunction in anhedonic patients, involving the reward system and basal ganglia, in particular ventral striatum (nucleus accumbens) [17,19,20]. On the other hand, no consideration regarding the potential relationship between hopelessness and dopaminergic dysfunction has been reported in previous research [21,22,23,24,25,26].

Considering the common psychopathological aspects shared by anhedonia and hopelessness, such as the loss of motivation and negative beliefs, which seems to involve the dopaminergic reward system, we hypothesized that hopelessness would be correlated to a striatal dopaminergic disfunction, as previously reported for anhedonia.

The inverse correlation between hopelessness intensity and DAT availability involving all the basal ganglia is an interesting element emerged in our study, since previous evidence on anhedonia showed that dopaminergic dysfunction appeared to be particularly focused in ventral and only in some specific areas of dorsal striatum (particularly caudate). From a clinical point of view, this is consistent with the substantial differences characterizing anhedonia and hopelessness.

The former represents the inability to experience pleasure or interest in almost all activities of daily life and seems to be a transdiagnostic dimension which is not only interesting for studies on MDD [37]. The latter consists of negative expectations about the future, which could involve the self and others, where subjects find it impossible to solve their problems, so that they never reach their life goals [3]. This lack of hope could lead to suicidal ideation as suicide becomes perceived as the only way to escape from a presumed tragic and incomplete future. 

In our opinion, these findings could be explained by considering the different dopaminergic circuits involved; while anhedonia seems to be principally related to a dysfunction of the ventral striatum (considered the main core of dopaminergic reward pathways), hopelessness seems to involve different and more complexly subcortical DA pathways. In particular, a dysfunction of the Default Mode Network (DMN) [38] that regulates mind wandering [39] and the ability to reflect on their own events [40] could lead to hyper-reflexivity and rumination [41] on a present that, for a depressed patient, appears impossible to live and endure [42]. In this context, hopelessness appears as an element generated by wider and deeper alterations of the dopaminergic pathways, generating clinical manifestations different from anhedonia.

Another important finding of our investigation was the positive correlation between dissociative symptoms and hopelessness: depressed patients with high hopelessness experience severe dissociative symptoms. From a clinical point of view, this finding could be explained when considering dissociation as a way to coexist with hopelessness: dissociative symptoms seem to be a manner to deal with stressful mental processes and tolerate the lack of positive future prospects and the emotional pain and distress experienced by these patients [12].

Concerning suicidality, our preliminary analysis shows differences between high suicide risk and low suicide risk MDD patients in DAT availability: patients with high suicidality showed lower DAT levels in right caudate and left putamen. These findings are particularly interesting considering that the role of dopaminergic dysregulations in suicidality is still relative uncertain [21,22,23,24,25,26].

Our results, therefore, highlighting a dopaminergic dysfunction of basal ganglia both in hopelessness and in suicidality separately, reinforce the psychopathological correlation of the two aforementioned clinical dimensions already described in previous studies [43].

Taken together, the finding of low levels of DAT in subcortical areas in subjects with severe hopelessness and high suicidality suggests the possible use of dopaminergic system modulators as strategies to contain suicidal risk in these patients. Analyzing patient responses to pharmacological agents modulating the dopaminergic system could improve the clinical outcomes and deserve to be specifically tested. These treatments are potentially effective in the context of integrated clinical approaches that include adequate psychotherapeutic management. Furthermore, considering our preliminary results, we can speculate that the treatment of high hopelessness of MDD patients through dopaminergic modulators could help physicians to contain dissociative symptoms, considering the frequent cooccurrence of hopelessness and dissociation in these patients.

## 5. Limitations

Our results should be interpreted cautiously due to several limitations: firstly, the small sample size could be an important limitation to our speculations. Secondly, ^123^I-FP-CIT is a non-specific radiotracer for DAT because of its capacity to bind the serotonin transporter (SERT), although only low-SERT density has been reported in the basal ganglia [44,45]. Thirdly, our study does not include a detailed assessment of suicidality through the use of a specific scale, since BHS gives only an indirect indication of patients’ suicidal risk and the 3rd item of HDRS-21 is not a validated scale for the assessment of suicidal risk. In addition, possible confounding factors (i.e., marital status, traumatic experiences, number of MDE, time from the first MDE) should be taken into account in future research, since they could affect DAT levels in basal ganglia. 

## 6. Conclusions

Our results support the hypothesis of specific pathophysiological alterations underpinning different MDD endophenotypes. In particular, the significant correlation between hopelessness and striatal dopaminergic dysfunction represents new and important evidence on this topic. Furthermore, the correlation between suicidality and striatal dopaminergic dysfunction is particularly intriguing, although our results should be confirmed with validated assessment scales for suicidality. Further studies on both hopelessness and suicidality are necessary to confirm our results and to test potential interventions to reverse the functional consequences caused by these leading clinical features of MDD. If confirmed, these results could help physicians treat patients by considering their peculiar clinical features and possibly provide dopaminergic treatments specific for hopelessness and prevent suicidal ideation.

## Figures and Tables

**Table 1:** Descriptive for the variables of the study (*n* = 51).

Variable	Number (Percentage)	Mean	SD	Range	Median
Age		65.82	12.42	30–82	69
Education years		10.6	5.10	5–18	10.5
Sex					
Male Female	22 (43.1%)				
	29 (56.1%)				
HDRS-21		21.67	3.51	18–31	21
Suicidality (HDRS-21 ITEM 3 > 0)	20 (39.2%)				
BHS		11.57	3.1	6–19	12
HAM-A		15.35	3.19	10–23	15
DES		7.42	6.06	1.07–38.93	6.07

*Note.* SD: standard deviation. HDRS-21: 21-item Hamilton Depression Rating Scale; BHS: Back Hopelessness Scale; SHAPS: Snaith Hamilton Pleasure Scale; HAM-A: Hamilton Anxiety Rating Scale; DES: Dissociative Experiences Scale.

**Table 2:** DAT striatal availability.

Striatal 123I-FP-CIT Binding Ratios	Mean	SD	Range	Median
Right Putamen	2.56	0.33	1.68–3.29	2.60
Left Putamen	2.46	0.37	1.54–3	2.52
Right Caudate	3.35	0.45	2.07–4.3	3.27
Left Caudate	3.29	0.40	2.39–4.1	3.30
Right Striatum	2.68	0.43	1.65–3.61	2.69
Left Striatum	2.63	0.43	1.72–3.56	2.59

*Note.* SD: standard deviation.

**Table 3:** DAT striatal availability in High suicide Risk (*n* = 20) vs. Low suicide Risk subjects (*n* = 31) (ANOVA).

Striatal 123I-FP-CIT Binding Ratios		Mean	SD	Range	F-value	*p* Value
Right Putamen	High Suicide Risk	2.48	0.36	1.68–3.29	1.598	0.212
	Low Suicide Risk	2.60	0.30	2.00–3.29		
Left Putamen	High Suicide Risk	2.31	0.45	1.54–3.00	5.677	0.021 *
	Low Suicide Risk	2.56	0.27	1.82–3.00		
Right Caudate	High Suicide Risk	3.19	0.50	2.07–4.30	4.412	0.041 *
	Low Suicide Risk	3.45	0.39	2.95–4.28		
Left Caudate	High Suicide Risk	3.18	0.43	2.39–3.90	2.918	0.094
	Low Suicide Risk	3.37	0.36	2.70–4.10		
Right Striatum	High Suicide Risk	2.58	0.43	1.65–3.40	1.633	0.207
	Low Suicide Risk	2.74	0.42	1.81–3.61		
Left Striatum	High Suicide Risk	2.52	0.44	1.76–3.56	2.150	0.149
	Low Suicide Risk	2.70	0.41	1.72–3.50		

Note. SD: standard deviation. *: *p* value < 0.05.

## Supplementary Materials

The following are available online at https://www.mdpi.com/2076-3425/10/8/519/s1, **Table S1:** correlation between hopelessness and DAT levels in basal ganglia, **Table S2:** correlations between psychometric scales, **Table S3:** correlation between psychometric scales and DAT levels in basal ganglia.
