# Supplementary material for: Hopelessness, Dissociative Symptoms, and Suicide Risk in Major Depressive Disorder: Clinical and Biological Correlates
Source: Brain Sci. 2020 Aug 5;10(8):519. doi: 10.3390/brainsci10080519 (PMC7465542; doi:10.3390/brainsci10080519)
Supplement: Supplementary file 1 [file brainsci-10-00519-s001.pdf]

Table S1. Correlation between hopelessness and DAT levels in basal ganglia.

				STDX	STSN	CADX	CASN	PUDX	PUSN
**BHS TOT**	**Pearson's correlation**			-,281*	-,317*	-,398**	-,467**	-,445**	-,454**
	**Sign. (two- tailed)**			,046	,024	,004	,001	,001	,001
	**N**			51	51	51	51	51	51
	**Bootstrap[c]**	**Distortion**		,005	,005	,008	,006	,007	,003
		**Deviation Error**		,153	,152	,119	,106	,105	,096
		**Confidence range 95%**	**Inferior**	-,565	-,591	-,600	-,649	-,618	-,633
			**Superior**	,043	,004	-,119	-,235	-,230	-,260

* Correlation is significant at level 0.05 (two-tailed); ** Correlation is significant at level 0.01 (two-tailed); c: Unless otherwise specified, bootstrap results are based on 1000 bootstrap samples. *Notes* BHS TOT: Total score of Beck Hopelessness Scale; STDX: Right Striatum; STSN: Left Striatum; CADX: Right Caudate; CASN: Left Caudate; PUDX: Right Putamen; PUSN: Left Putamen.

Table S2. Correlations between psychometric scales.

		HAMATOT	HAMDTOT	BHSTOT	DESTOT
**HAM-A TOT**	**Pearson's correlation**	1	,532**	,048	,194
	**Sign. (two-tailed)**		,000	,738	,174
	**N**	51	51	51	51
**HAM-D TOT**	**Pearson's correlation**	,532**	1	,371**	,386**
	**Sign. (two-tailed)**	,000		,007	,005
	**N**	51	51	51	51
**BHS TOT**	**Pearson's correlation**	,048	,371**	1	,320*
	**Sign. (two-tailed)**	,738	,007		,022
	**N**	51	51	51	51
**DES TOT**	**Pearson's correlation**	,194	,386**	,320*	1
	**Sign. (two-tailed)**	,174	,005	,022	
	**N**	51	51	51	51

* Correlation is significant at level 0.01 (two-tailed); ** Correlation is significant at level 0.05 (two-tailed). *Notes* HAM-A TOT: Total score of Hamilton Anxiety Rating Scale; HAM-D TOT: Total score of Hamilton

Depression Rating Scale; BHS TOT: Total score of Beck Hopelessness Scale; DES TOT: Total score of Dissociative Experiences Scale.

Table S3. Correlation between psychometric scales and DAT levels in basal ganglia.

				STDX	STSN	CADX	CASN	PUDX	PUSN
**HAM-A TOT**	**Pearson's correlation**			-,063	-,039	-,158	-,001	-,010	-,119
	**Sign. (two- tailed)**			,660	,786	,267	,995	,945	,405
	**N**			51	51	51	51	51	51
	**Bootstrap[c]**	**Distortion**		,007	,005	,007	,006	,007	,013
		**Deviation Error**		,116	,113	,122	,137	,123	,139
		**Confidence range 95%**	**Inferior**	-,292	-,276	-,395	-,264	-,242	-,369
			**Superior**	,171	,176	,093	,270	,240	,187
**DES TOT**	**Pearson's correlation**			-,099	-,093	-,220	-,189	-,163	-,153
	**Sign. (two- tailed)**			,491	,514	,121	,183	,254	,284
	**N**			51	51	51	51	51	51
	**Bootstrap[c]**	**Distortion**		,023	,019	,026	,007	,014	,020
		**Deviation Error**		,162	,154	,159	,124	,148	,163
		**Confidence range 95%**	**Inferior**	-,352	-,346	-,443	-,391	-,407	-,394
			**Superior**	,276	,255	,180	,085	,177	,228
**HAMD TOT**	**Pearson's correlation**			-,237	-,196	-,388**	-,227	-,186	-,291*
	**Sign. (two- tailed)**			,094	,169	,005	,109	,190	,038
	**N**			51	51	51	51	51	51
	**Bootstrap[c]**	**Distortion**		,010	,007	,014	,011	,011	,004
		**Deviation Error**		,142	,131	,131	,136	,162	,151
		**Confidence range 95%**	**Inferior**	-,497	-,432	-,601	-,466	-,476	-,571
			**Superior**	,059	,077	-,099	,059	,136	,011

* Correlation is significant at level 0.01 (two-tailed); ** Correlation is significant at level 0.05 (two-tailed); c: Unless otherwise specified, bootstrap results are based on 1000 bootstrap samples. *Notes* HAM-A TOT: Total score of Hamilton Anxiety Rating Scale; DES TOT: Total score of Dissociative Experiences Scale.
